# Commentary: Why Haven’t We Found an Effective Treatment for COVID-19?

**DOI:** 10.3389/fimmu.2021.714175

**Published:** 2021-07-05

**Authors:** Josef Brzoska, Harald von Eick, Manfred Hündgen

**Affiliations:** Retired, Laupheim, Germany

**Keywords:** COVID-19, coronavirus, interferons - pharmacology, therapeutic use, corticosteroids, clinical trials

## Introduction

In their article, Spicer and Jalkanen (1) point out that the search for a single agent to treat COVID-19 is an inappropriate approach. They emphasize that the phase of this disease determines which drug can be therapeutically effective. Patients benefit from the administration of antivirals, such as interferons, only in the early phase of this disease. In the late phase, treatment strategies should be aimed to suppress the cytokine storm. Accordingly, only then can corticosteroids be reasonably administered. In a short phase in-between, a targeted therapy can be applied. Harmful drug interactions have to be avoided, especially between glucocorticoids and interferons, because glucocorticoids can block interferon signalling pathways. Spicer and Jalkanen (1) do not mention the dosages to be used with the different agents. However, these are decisive for treatment success in COVID-19. The use of the most appropriate glucocorticoid dose for suppressing the exaggerated inflammation bears the risk of an increased viral replication. Dexamethasone at a dose of 6 mg given once daily for up to 10 days reduces mortality only in hospitalized patients requiring respiratory support but tends to be harmful in COVID-19 patients with less severe disease (2). Diagnostic markers for the selection and adjustment of the glucocorticoid dosage still have to be determined further. There are also open issues regarding the therapeutic regimen to be used with interferons.

## Dosages of Interferons Used in COVID-19

Clinical studies in COVID-19 or other severe coronavirus infections have often provided disappointing results regarding the therapeutic efficacy of interferons (3–8). In the trials with systemic administration, interferon alfa and lambda were used as in chronic viral hepatitis, i.e., 3 to 10 million IU three times a week (or an equivalent dose of the PEGylated forms given once weekly) by the subcutaneous route. For the treatment with interferon beta, the dosage chosen was as in multiple sclerosis, i.e., 8 or 12 million IU, respectively, thrice weekly by the subcutaneous route. Dose-finding studies with interferons in COVID-19 have not been performed. Several explanations for the failure of interferons in this disease have been provided (4, 5), often not considering an inappropriate therapeutic regimen, which we think is one critical factor (3). Only recently, others also suspect a too low dosage and/or a less effective route of administration as potential reasons for the failure of interferons in COVID-19 (7, 8). For interferon beta, Jalkanen et al. (9) emphasize the importance of an intravenous administration. However, their recommended daily dosage of 10 µg (ca. 2.5 million IU) given as a short-time infusion was neither successful in acute respiratory distress syndrome (10) nor in COVID-19 (11). In our opinion, this dosage and infusion time is not sufficient for interferon beta-1a in acute severe viral diseases. Instead, another therapeutic regimen should be employed according to clinical studies performed about 40 years ago but, apparently, not generally known anymore (3).

## Early Trials With Interferons in Acute Viral Diseases

In 1978, the results of three placebo-controlled studies in herpes zoster treated with native interferon alpha were published (12). In these trials, only the highest dose (0.51 million IU per kg body weight, i.e., 36 million IU in a patient with a body weight of 70 kg) given daily by intramuscular injections for 5 to 7 days and thus leading to a continuous high serum level was therapeutically effective in contrast to lower daily doses (12 million IU or 3 million IU, respectively, for a patient weighing 70 kg). However, this form of high-dose interferon therapy was not pursued any longer since acyclovir received approval for the treatment of herpes zoster in the early 1980s. It was disregarded that the dosage determined for an effective treatment with interferon in herpes zoster could also be indicative for other severe acute viral infections. This approach was only continued with native interferon beta in Germany until the 2000s (3). Because of its distinct tissue affinity, high-dose native interferon beta (25 million IU in an adult) was administered daily for 3 to 5 consecutive days *via* the intravenous route as a continuous infusion to achieve sufficiently high and persistent serum levels. A daily infusion time of less than 24 h and lower doses revealed no or only slight therapeutic effects. The potent regimen determined in herpes zoster was also successfully employed to treat other severe acute viral infections (e.g., viral encephalitis) with native interferon beta (3). It has to be emphasized that such a high-dose treatment was typically associated with severe but transient side effects requiring hospitalization under intensive care conditions.

## Dosages to Be Used With Interferons in COVID-19

Accordingly, we believe that also in COVID-19 a high or the maximum tolerated dose (MTD), respectively, should be given daily for ca. 5 consecutive days if interferon is administered systemically as an antiviral monotherapy (3). However, the MTD of most interferon products currently available has still to be determined, in particular regarding long-term intravenous administration of interferon beta. In COVID-19, a lower dosage than the MTD (as well as the subcutaneous route for interferon beta) can be therapeutically effective only if synergistic effects are utilized, i.e., if interferon is used in combination with other antiviral agents, as demonstrated in some recent clinical studies (13–16). Furthermore, relatively low doses can successfully be employed without concomitant administration of other antivirals if interferon is given by inhalation (17, 18). However, interferon applied by this route will probably act on the (upper) respiratory tract only but not on other organs, which are frequently infected by SARS-CoV-2, too, especially in severe cases of COVID-19.

## Discussion

Several studies have shown that timing of interferon administration is one decisive factor in COVID-19 (13–16). A positive treatment effect can be achieved only if interferon is used early in the course of this disease. Furthermore, a therapeutically effective regimen (with a sufficient dose of interferon and an appropriate route of administration) has to be chosen. Simultaneous use of glucocorticoids should be avoided because of the abovementioned reasons. Only if all these aspects are considered, interferon therapy can be successful in COVID-19.

## Author Contributions

JB made the literature search and wrote the first draft of the manuscript. This draft was intensively discussed with the two other authors and changed according to their comments. All authors contributed to the article and approved the submitted version.

## Conflict of Interest

The authors declare that the research was conducted in the absence of any commercial or financial relationships that could be construed as a potential conflict of interest.
